# A single-component multidrug transporter of the major facilitator superfamily is part of a network that protects *E scherichia coli* from bile salt stress

**DOI:** 10.1111/mmi.12597

**Published:** 2014-04-13

**Authors:** Stephanie Paul, Kamela O Alegre, Scarlett R Holdsworth, Matthew Rice, James A Brown, Paul McVeigh, Sharon M Kelly, Christopher J Law

**Affiliations:** 1 Institute for Global Food Security, School of Biological Sciences, Medical Biology Centre, Queen’s University Belfast97 Lisburn Road, Belfast, BT9 7BL, UK; 2 Institute of Molecular Cell and Systems Biology, College of Medical, Veterinary and Life Sciences, University of GlasgowGlasgow, G12 8QQ, UK

## Abstract

Resistance to high concentrations of bile salts in the human intestinal tract is vital for the survival of enteric bacteria such as *E scherichia coli*. Although the tripartite AcrAB–TolC efflux system plays a significant role in this resistance, it is purported that other efflux pumps must also be involved. We provide evidence from a comprehensive suite of experiments performed at two different pH values (7.2 and 6.0) that reflect pH conditions that *E . coli* may encounter in human gut that MdtM, a single-component multidrug resistance transporter of the major facilitator superfamily, functions in bile salt resistance in *E . coli* by catalysing secondary active transport of bile salts out of the cell cytoplasm. Furthermore, assays performed on a chromosomal Δ*acrB* mutant transformed with multicopy plasmid encoding MdtM suggested a functional synergism between the single-component MdtM transporter and the tripartite AcrAB–TolC system that results in a multiplicative effect on resistance. Substrate binding experiments performed on purified MdtM demonstrated that the transporter binds to cholate and deoxycholate with micromolar affinity, and transport assays performed on inverted vesicles confirmed the capacity of MdtM to catalyse electrogenic bile salt/H^+^ antiport.

## Introduction

The mammalian intestine is a complex, densely populated microbial ecosystem that is home to hundreds of different species and strains of bacteria (Ley *et al*., 2006; Qin *et al*., 2010; De Paepe *et al*., 2011). To survive and proliferate in, for example, the human gastrointestinal tract, the enteric bacterium *Escherichia coli* has evolved not only to resist the acidic environment of the duodenum but also the antimicrobial effects of up to 30 mM concentrations of bile salts and their free acids (Thanassi *et al*., 1997; Kalantzi *et al*., 2006; Merritt and Donaldson, 2009; Darkoh *et al*., 2010). Bile salts are amphipathic, water-soluble, steroidal surfactants, synthesized in the liver from cholesterol and secreted into the bile, that aid emulsification and enzymatic digestion of dietary lipids in the small intestine (Maldonado-Valderrama *et al*., 2011). In humans, bile acids are secreted from hepatocytes as salts, in a form conjugated, via an amide bond, to glycine or taurine (Maldonado-Valderrama *et al*., 2011). Although the majority of these bile salts are reabsorbed by the lining of the ileum for subsequent reuse, those that remain are deconjugated by bacterial enzymes to form free primary bile acids such as cholate (Hofmann, 1999; Ridlon *et al*., 2006). Further reactions, again catalysed by intestinal flora enzymes, transform the primary bile acids into the secondary bile acids deoxycholate (DOC) and lithocholate (Hylemon *et al*., 1991; Hofmann, 1999; Ridlon *et al*., 2006); the latter is rapidly eliminated from the body and constitutes only trace amounts of the unconjugated biliary bile acids, the remainder being about equal amounts of cholate, chenodeoxycholate and deoxycholate (Hofmann, 1999).

Over the physiological pH range encountered in the small intestine, conjugated bile salts are fully ionized, and are therefore present as strongly acidic bile acid anions (Hofmann, 1999) that require the OmpF porin in order to traverse the outer membrane of Gram-negative bacteria (Thanassi *et al*., 1997). In contrast, the unconjugated bile salts are weakly acidic molecules that, in their uncharged, protonated state, can diffuse across both the outer and inner membranes of Gram-negative bacteria to accumulate in the cell cytoplasm from where they exert their cytotoxic effects by way of disruption of cell membrane integrity, promotion of RNA secondary structure formation, DNA damage, denaturation of cellular proteins, and oxidative stress (Merritt and Donaldson, 2009). Therefore, *E. coli* and other enteric bacteria must possess mechanisms to defend against these types of injury. The main defensive mechanism employed by Gram-negative bacteria is the active extrusion of bile salts and their derivatives from the interior of the cell by multidrug resistance efflux systems (Gunn, 2000). Intrinsic resistance to bile salts in *E. coli* is conferred in part by the constitutively expressed resistance-nodulation-division (RND)-type and major facilitator superfamily (MFS)-type tripartite AcrAB–TolC and EmrAB–TolC multidrug efflux systems respectively (Ma *et al*., 1995; Fralick, 1996; Thanassi *et al*., 1997). More minor contributions are provided by YdhE of the multidrug and toxic compound extrusion (MATE) family, and the YdgEF small multidrug resistance (SMR) protein (Nishino and Yamaguchi, 2001). However, it has been posited that an additional, unidentified efflux system, probably a proton antiporter, also plays a significant role in protecting *E. coli* from the noxious effects of bile salts (Thanassi *et al*., 1997).

Apart from those proteins already confirmed as playing a role in bile salt efflux, the *E. coli* ‘effluxome’ contains 33 other putative multidrug transporters that, considering the broad catalogue of chemically and structurally dissimilar substrates they can handle, are candidates to function in this role (Nishino and Yamaguchi, 2001). Our previous work on one of these transporters, MdtM, suggested it might possess capacity to function physiologically in removal of bile salts from the cell (Holdsworth and Law, 2012a, b). MdtM is a single-component, 12 transmembrane-spanning drug/H^+^ antiporter of the DHA1 subfamily of the MFS (Holdsworth and Law, 2012a) that is energized by components of the transmembrane electrochemical gradient (Holdsworth and Law, 2013). MdtM is extremely versatile with respect to function; apart from its role as a multidrug transporter that can efflux several classes of antibiotic and a variety of quaternary ammonium compounds from the cell cytoplasm (Edgar and Bibi, 1997; Nishino and Yamaguchi, 2001; Tal and Schuldiner, 2009; Soo *et al*., 2011; Holdsworth and Law, 2012a, b), it also possesses a Na^+^(K^+^)/H^+^ antiport activity that enables it to function in alkaline pH homeostasis in *E. coli* (Holdsworth and Law, 2013). Although a putative *Eubacterium* MFS transporter, BaiG, has been implicated in the uptake of bile acids (Mallonee and Hylemon, 1996), a role for single-component transporters of the MFS in their efflux has not been reported. We performed growth inhibition studies, transport assays with inverted membrane vesicles, and substrate binding studies using purified protein to investigate if MdtM confers *E. coli* with resistance against the harmful effects of cholate and deoxycholate (Doerner *et al*., 1997) at two different physiologically relevant pH values: (i) at a pH of 7.2 that reflects the pH of the colon (McDougall *et al*., 1993), and (ii) at a pH of 6.0 that reflects the acidic pH of the duodenum (Rune and Viskum, 1969). Taken together, the results of our experiments support MdtM as part of the efflux network that protects *E. coli* from bile salt stress; this activity likely represents a natural physiological role of the protein (Neyfakh, 1997; Piddock, 2006).

## Results

### Bile salt resistance in *E . coli* is aided by *mdtM*

To test for a contribution by the *mdtM* gene product to bile salt resistance in *E. coli*, the growth of wild-type *E. coli* BW25113 and its isogenic Δ*mdtM* mutant under cholate and deoxycholate stress was investigated by measuring the minimum inhibitory concentration (MIC) of each bile salt (Table 1). The BW25113 strain of *E. coli* was chosen for this aspect of the study as it contains a full complement of multidrug efflux proteins and has been used before for investigations into MdtM function (Tal and Schuldiner, 2009; Holdsworth and Law, 2012a, b; 2013). MICs of cholate and deoxycholate for *E. coli* BW25113 single-deletion mutants that are dysfunctional with respect to tripartite EmrAB–TolC and AcrAB–TolC efflux activity were also determined to permit a comparison of the contribution of each of those transporters to bile salt resistance. Additionally, the effect of deleting the outer membrane porin *ompF* in the BW25113 strain was tested.

**Table 1:** MICs of cholate and deoxycholate in isogenic strains of *E . coli* BW25113

Strain	MIC (mg ml^−1^) of	
	Na^+^ cholate	Na^+^ deoxycholate
Wild-type	128	128
Δ*mdtM*	64	32
Δ*acrB*	8	< 1
Δ*emrB*	128	64
Δ*ompF*	128	128

As shown in Table 1, deletion of *mdtM* resulted in a phenotype that was twofold more susceptible to cholate and fourfold more susceptible to deoxycholate than wild-type *E. coli* BW25113. Not surprisingly, considering the especially significant role of the tripartite efflux system AcrAB–TolC in bile salt resistance in *E. coli* (Ma *et al*., 1995; Thanassi *et al*., 1997), deletion of chromosomal *acrB* resulted in a bile salt-hypersensitive phenotype with cholate and deoxycholate MICs decreased from 128 to 8 mg ml^−1^ and from 128 to < 1 mg ml^−1^ respectively. The difference in sensitivity to each bile salt is a consequence of their hydrophobicity (which is highest for deoxycholate), and hence, with the ability of each to diffuse across the bacterial membrane system and into the cell cytoplasm (Kamp *et al*., 1993). The ability of the unconjugated bile salts to diffuse freely across the bacterial membranes was further illustrated by the MICs determined in the Δ*ompF* mutant; deletion of OmpF porin in the BW25113 strain had no effect on bile salt resistance.

In contrast to the dramatic effect that deletion of chromosomal *acrB* had on bile salt resistance, the absence of a functional MFS-type EmrAB–TolC tripartite efflux system had negligible effect on cholate resistance, and resistance to deoxycholate decreased only twofold. The apparent lack of protection against cholate by a system that reportedly plays a significant role in bile salt resistance (Thanassi *et al*., 1997; Li and Nikaido, 2004) is not unexpected because the AcrAB–TolC and EmrAB–TolC tripartite pumps work in parallel and not in series, and therefore their effect is additive and not multiplicative (Lee *et al*., 2000). As such, the contribution of EmrAB–TolC is much smaller as compared to AcrAB–TolC and the effect of *emrB* deletion is seen only in the background of dysfunctional AcrAB–TolC (Thanassi *et al*., 1997).

The MIC determinations suggest that, although not essential, the *mdtM* gene product makes a significant contribution to both cholate and deoxycholate resistance in *E. coli*. Analysis of *mdtM* transcript levels revealed that they are not upregulated upon bile salt challenge and that there was no statistically significant change in *mdtM* transcription between cholate-treated *versus* untreated control *E. coli*, relative to a 16S rRNA reference (data not shown). This implies that the ‘housekeeping’ levels of MdtM present in the membrane at the onset of bile salt challenge are sufficient to contribute effectively to resistance.

### Expression of plasmidic *mdtM* recovers a bile salt-resistant phenotype

To test further the contribution of *mdtM* to bile salt resistance, Δ*mdtM* cells were transformed with an arabinose-inducible multicopy plasmid (pMdtM) that encoded wild-type MdtM, and the ability of overexpressed transporter to protect cells from the cytotoxic effects of cholate and deoxycholate was determined by growth inhibition assays that measured the concentration of bile salt required to inhibit cell growth by 50% of that measured in the absence of the substrate (the IC_50_ value) (Soothill *et al*., 1992). Δ*mdtM* cells that overproduced dysfunctional MdtM from the pD22A plasmid acted as a control (Holdsworth and Law, 2012a). Assays were performed in LB liquid media buffered to two different pH values (7.2 and 6.0) that mirror the pH conditions experienced by enteric bacteria as they negotiate the mammalian intestine (Rune and Viskum, 1969; McDougall *et al*., 1993).

**Figure 1:**
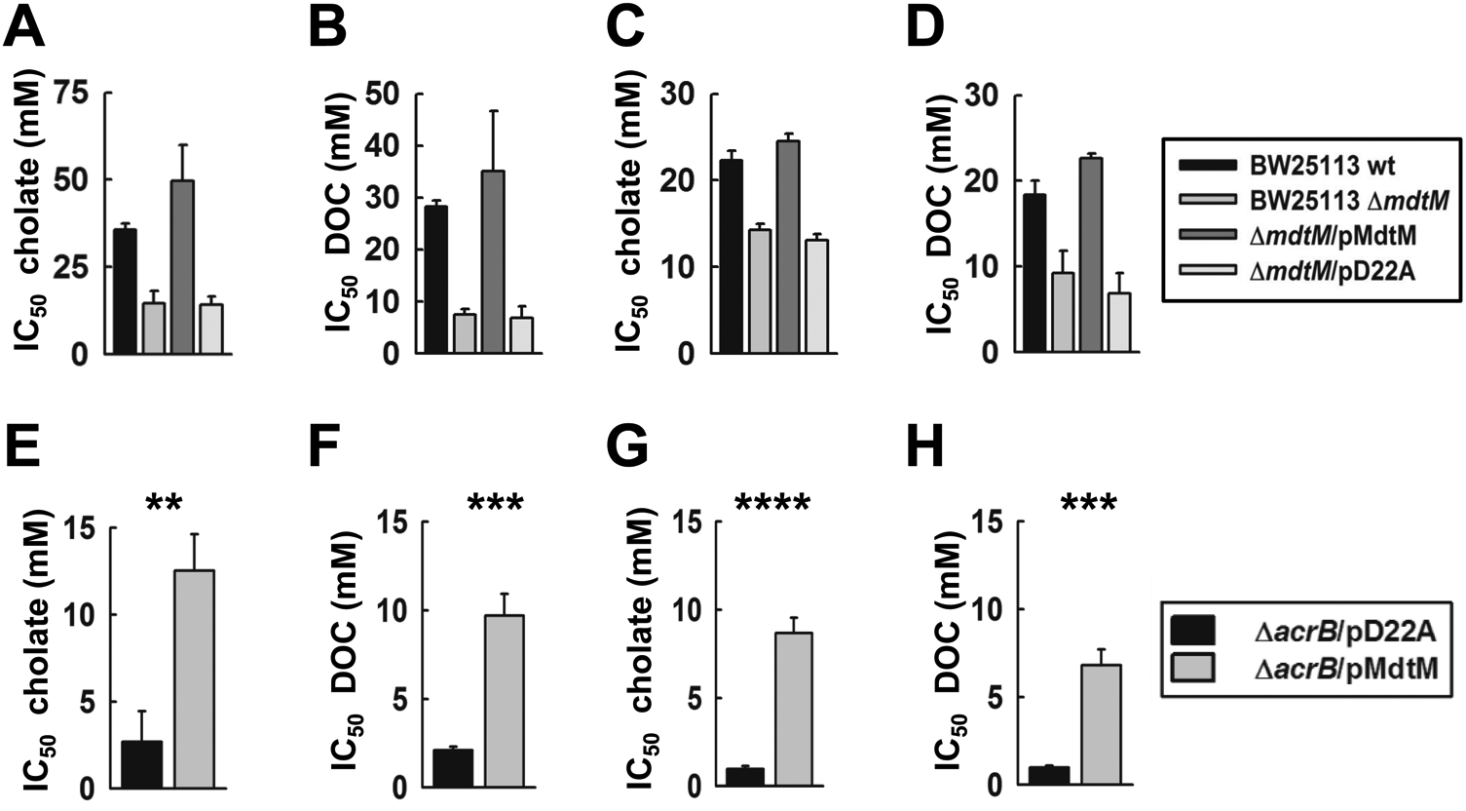
Susceptibility of *E . coli* BW25113 wild-type cells and Δ*mdtM* single-deletion mutant, and Δ*mdtM* and Δ*acrB* single-deletion mutants transformed with pMdtM or pD22A to bile salts.A–D. IC_50_ measurements revealed that transformation of *E. coli* BW25113 Δ*mdtM* cells with plasmidic *mdtM* enhanced bile salt resistance. IC_50_ of (A) cholate at pH 7.2; (B) deoxycholate at pH 7.2; (C) cholate at pH 6.0; (D) deoxycholate at pH 6.0.E–H. The Δ*acrB* chromosomal deletion mutant that overexpressed dysfunctional MdtM D22A mutant was hypersensitive to bile salts. IC_50_ of (E) cholate at pH 7.2; (F) deoxycholate at pH 7.2; (G) cholate at pH 6.0; (H) deoxycholate at pH 6.0.Bars and error bars represent the mean ± SD of three separate experiments. **Significant at *P* < 0.01; ***significant at *P* < 0.001; ****significant at *P* < 0.0001.

As shown in Fig. 1A–D, deletion of chromosomal *mdtM* resulted in a phenotype that was clearly more susceptible to bile salts than wild-type cells. The data also revealed the BW25113 strain of *E. coli* to be less resistant to deoxycholate than to cholate; at the slightly alkaline pH of 7.2, the IC_50_ value of wild-type BW25113 for cholate was 35.6 ± 1.8 mM, whereas an IC_50_ of 28.2 ± 1.2 mM was calculated for the same cell type grown in the presence of deoxycholate (Fig. 1A and B); these IC_50_ values are about threefold and fourfold greater, respectively, than the IC_50_ values (14.6 ± 3.6 mM for cholate, and 7.5 ± 1.1 mM for deoxycholate) calculated for Δ*mdtM* single-deletion mutant cells grown under the same conditions. However, complementation of Δ*mdtM* cells with plasmidic *mdtM* (pMdtM) recovered a phenotype with greater than wild-type levels of resistance to each bile salt. In contrast, Δ*mdtM* control cells that overexpressed dysfunctional MdtM D22A mutant transporter from a multicopy plasmid (pD22A) displayed no resurrection of bile-salt resistance.

Comparison of the IC_50_ data also revealed that susceptibility of wild-type cells to both bile salts was enhanced at acidic pH, with IC_50_ values of 22.3 ± 1.1 mM and 18.4 ± 1.6 mM for cholate and deoxycholate, respectively, for cells grown at pH 6.0 (Fig. 1C and D). The IC_50_ values of the Δ*mdtM* mutant cells and those that overexpressed dysfunctional MdtM at this pH, however, were very similar to those calculated for the same cells grown at pH 7.2. Again, overexpression by Δ*mdtM* cells of functional MdtM from a multicopy plasmid rescued a bile salt-resistant phenotype. In all cases, statistical analysis of the IC_50_ data using a two-tailed Student’s *t*-test confirmed that the differences in susceptibility observed between wild-type and Δ*mdtM* mutant cells to the bile salts under test were significant (*P* < 0.05). Western blot analysis of dodecyl-β-d-maltopyranoside (DDM) detergent-solubilized cytoplasmic membranes from Δ*mdtM* cells transformed with pMdtM or pD22A provided evidence that the observed differences in susceptibility to the bile salts did not originate from differences in the expression of recombinant transporter; the levels of wild-type MdtM and dysfunctional D22A protein production in the Δ*mdtM* transformants grown at pH 7.2 and pH 6.0 were similar (Fig. S1A).

### MdtM acts synergistically with AcrAB–TolC to efflux bile salts

Evidence suggests that single-component membrane transporters can interact synergistically with tripartite RND-type proteins to form a dual-stage efflux mechanism that provides multiplicative effects on drug resistance (Lee *et al*., 2000). To test if interplay between MdtM and AcrAB–TolC holds for efflux of bile salts, and also if MdtM is capable of functioning unilaterally in bile salt efflux, we performed growth assays that measured the IC_50_ of cholate and deoxycholate for BW25113 Δ*acrB* cells transformed with plasmidic DNA encoding wild-type MdtM or, as a control, the dysfunctional D22A mutant (Fig. 1E–H). Levels of overproduction of wild-type and mutant MdtM protein were similar at both pH values investigated, as determined by Western blot analysis (Fig. S1B).

Absence of functional AcrAB–TolC and MdtM efflux systems resulted in cells that were hypersensitive to the cytotoxic effects of each bile salt, with IC_50_ values that were at least an order of magnitude smaller than the IC_50_s of wild-type cells (Fig. 1E–H). Complementation of Δ*acrB* cells with pMdtM recovered a phenotype that was less susceptible; at pH 7.2, the IC_50_ values of these cells for cholate and deoxycholate were 12.5 ± 2.1 mM and 9.7 ± 1.2 mM, respectively (Fig. 1E and F), and at pH 6.0 the corresponding IC_50_ values were 8.7 ± 0.9 mM and 6.8 ± 0.9 mM (Fig. 1G and H). Although these IC_50_ values were approximately four- to fivefold greater than those of the Δ*acrB* mutant transformed with pD22A (Fig. 1E–H), they were similar to those measured for the chromosomal *mdtM*-deletion mutant under the same conditions (Fig. 1A–D). The inability of Δ*acrB* cells that overproduced MdtM to recover the levels of cholate and deoxycholate resistance observed in wild-type cells highlights the major role in efflux played by AcrAB–TolC and suggests that, even though MdtM clearly possesses capacity to function independently in bile salt resistance, a functional synergism between MdtM and AcrAB–TolC provides greater levels of resistance.

### Bile salts compete with other antimicrobials for MdtM-mediated efflux

Although the results of growth inhibition assays implicated the *mdtM* gene product in protecting cells from bile salt cytotoxicity, they did not provide any direct evidence that MdtM mediates bile salt efflux. To provide initial confirmation of this, the effect of the addition of sodium cholate and sodium deoxycholate on the ethidium bromide (EtBr) efflux activity of MdtM was measured by whole-cell fluorescence assays that used *E. coli* UTL2 outer permeability mutant cells transformed with plasmidic MdtM. These assays were performed at pH 6.0 to abrogate any potential interference by the electrogenic Na^+^/H^+^ antiport activity of MdtM that is apparent at pH values > 7.0 (Holdsworth and Law, 2013).

**Figure 2:**
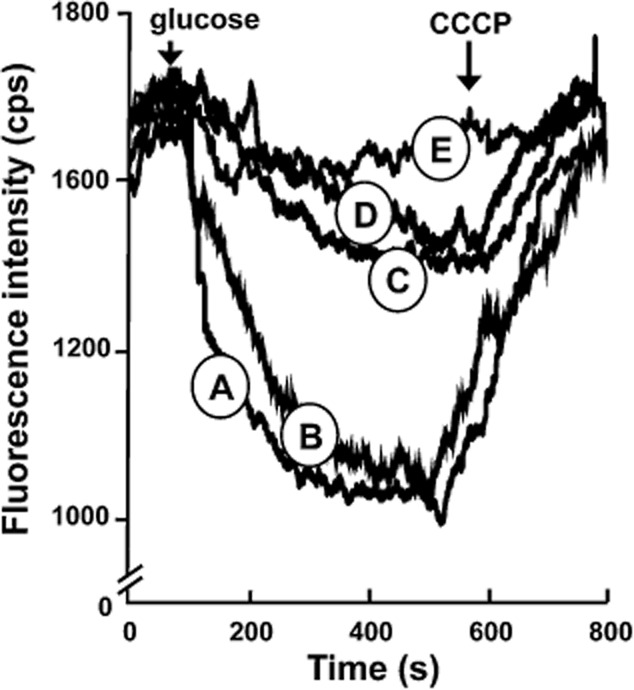
Whole-cell ethidium bromide transport assays. Both cholate and deoxycholate inhibited MdtM-mediated ethidium bromide (EtBr) efflux from *E . coli* UTL2 cells expressing recombinant wild-type MdtM. Traces represent EtBr efflux (A) in the absence of bile salt; (B) in the presence of 3 mM Na^+^; (C) in the presence of 3 mM sodium cholate; and (D) in the presence of 3 mM sodium deoxycholate. (E) A negative control was provided by UTL2 cells that expressed the dysfunctional MdtM D22A mutant. After 90 s in the fluorometer, cells loaded with EtBr were energized by addition of glucose (first arrow) and efflux of EtBr was monitored for a further 450 s. At this point, 100 μM CCCP was added as indicated (second arrow) to abolish active transport and the fluorescence emission was monitored for a further 260 s. All assays were performed at pH 6.0. Fluorescence intensity was measured in counts per second (cps).

In the positive control assay, performed in a buffer system that contained no added bile salt, addition of glucose to energize cells that expressed wild-type MdtM resulted in a rapid decrease in the fluorescence signal as the transporter actively extruded EtBr from the cell against its concentration gradient (Fig. 2, trace A). Subsequent addition of the ionophore carbonyl cyanide 3-chlorophenylhydrazone (CCCP) caused the fluorescence signal to rise again, indicating that the observed EtBr efflux was driven by the electrochemical gradient. Negative control cells that expressed dysfunctional MdtM D22A mutant from a multicopy plasmid were incapable of mediating any significant EtBr efflux (Fig. 2, trace E). To ensure that any observed inhibition of EtBr efflux was not due to competition by the sodium counterions of each added bile salt, transport experiments were performed with 3.0 mM NaCl added to the system (Fig. 2, trace B). Incubation of cells that overexpressed wild-type MdtM with sodium cholate or sodium deoxycholate at a final concentration of 3.0 mM prior to addition of glucose caused a dramatic decrease in the magnitude of the observed fluorescence signal that was indicative of inhibition of MdtM-mediated EtBr efflux by the bile salts (Fig. 2, traces C and D respectively). Subsequent collapse of the membrane potential by addition of CCCP resulted in a dequench of the fluorescence signal and suggested that detergent activity of the bile salts had not compromised inner membrane integrity. When re-energized, the same cells retained capacity to efflux EtBr (Fig. S2), adding further support to our contention that the reduction in EtBr transport activity observed in bile salt-treated cells was due to competition between the different substrates for MdtM and not due to perturbation of the cell membrane. In contrast, the effects of membrane disruption on EtBr transport were readily apparent from the results of control assays performed on cells treated with DDM detergent, a non-substrate of MdtM. In this case, addition of DDM to the assay mixture resulted in an immediate rise in the fluorescence signal as EtBr leaked back into the cell through the detergent-compromised membrane (Fig. S2).

### MdtM binds cholate and deoxycholate at micromolar concentrations

To confirm that MdtM was capable of binding bile salts, the binding of cholate and deoxycholate to purified transporter in dodecyl-β-d-maltoside (DDM) detergent solution at pH 6.0 and pH 7.2 was determined using intrinsic tryptophan (Trp) fluorescence with excitation at 295 nm. Sequential addition of bile salt aliquots to purified protein in DDM solution resulted in a concentration-dependent quenching of the Trp fluorescence emission signal without shifting the λ_max_ at 335 nm, suggesting that the quench resulted from a specific interaction between bile salt and the MdtM substrate-binding pocket, and that one or more Trp residues acted as reporters for binding. Threading of the MdtM primary sequence onto the 3-D crystal structure of the closely related *E. coli* MFS multidrug transporter EmrD (Yin *et al*., 2006) proposed only one of the nine Trp residues of MdtM, Trp309, as being located near the putative binding pocket of the transporter and, therefore, likely to be involved in the bile salt-induced quenching. Fluorescence measurements performed on Trp309Phe (W309F) and Trp309Ala (W309A) MdtM mutants supported this contention, with quenching induced by bile salt in both mutants severely reduced compared to that of the wild-type transporter and due probably to a non-specific interaction with the protein (Fig. S3).

**Figure 3:**
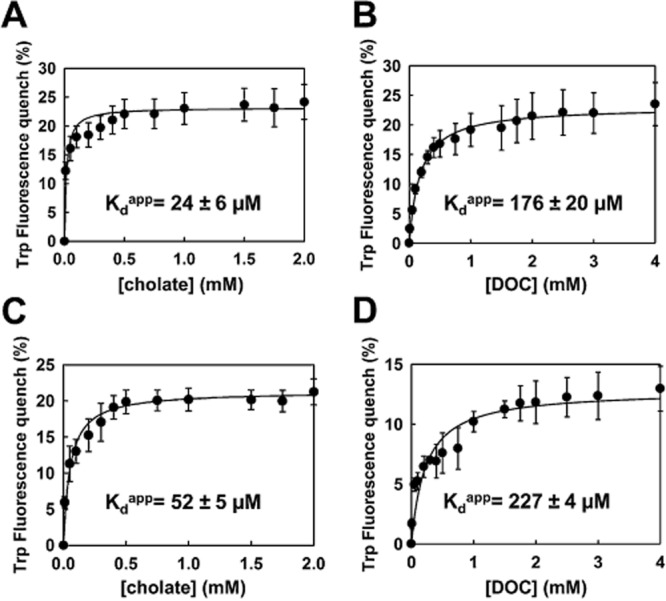
Substrate binding assays. Substrate-binding affinity curves showing percentage intrinsic fluorescence quenching of purified wild-type MdtM in DDM solution as a function of bile salt concentration. Binding affinity of (A) sodium cholate and (B) sodium deoxycholate for MdtM at pH 7.2, and (C) sodium cholate and (D) sodium deoxycholate for MdtM at pH 6.0. Curves were fitted using SigmaPlot 10. Data points and error bars represent the mean ± SD of three individual measurements.

Analysis of the binding data revealed that MdtM possessed micromolar affinity for the bile salts. The protein displayed greater affinity for cholate than deoxycholate and, as pH decreased, there was a concomitant decrease in binding affinity (Fig. 3). There was also a substrate- and pH-dependent variability in the magnitude of the Trp fluorescence quench, which ranged from about 25% in the presence of cholate at pH 7.2 (Fig. 3A) to about 12% in the presence of deoxycholate at pH 6.0 (Fig. 3D). At pH 7.2, the apparent dissociation constant (K_d_^app^) of MdtM for cholate was 24 ± 6 μM (Fig. 3A), whereas the protein appeared to bind deoxycholate about sevenfold less tight with a K_d_^app^ of 176 ± 20 μM (Fig. 3B). Acidic pH decreased the affinity of MdtM for both bile salts; at pH 6.0 the K_d_^app^ values for cholate and deoxycholate were 52 ± 5 μM (Fig. 3C) and 227 ± 4 μM (Fig. 3D) respectively. To ensure the observed increase in K_d_^app^ at acidic pH was not due to loss of structural integrity of the protein, the CD spectrum of purified transporter in DDM detergent solution at pH 6.0 and pH 7.2 was measured. Figure S4 illustrates the similarity in the CD spectra of MdtM at both pH values. The spectra are superimposable down to ∼ 196 nm, suggesting that there were no gross structural changes in the secondary structure of the protein following the change in pH. Below 196 nm there was a slight (< 10%) decrease in ellipticity in the spectrum of the transporter at the acidic pH (Fig. S4, black trace). The secondary structure estimates at each pH, however, were in close agreement (Table 2).

**Table 2:** Secondary structure content of detergent-solubilized MdtM at pH 7.2 and pH 6.0

pH	% α-helices	% β-sheets	% turns	% unknown
6.0	52.4	9.5	14.8	23.3
7.2	53.9	10.4	12.8	22.9

### MdtM mediates a bile salt/*H*^+^ exchange driven by the electrochemical gradient

**Figure 4:**
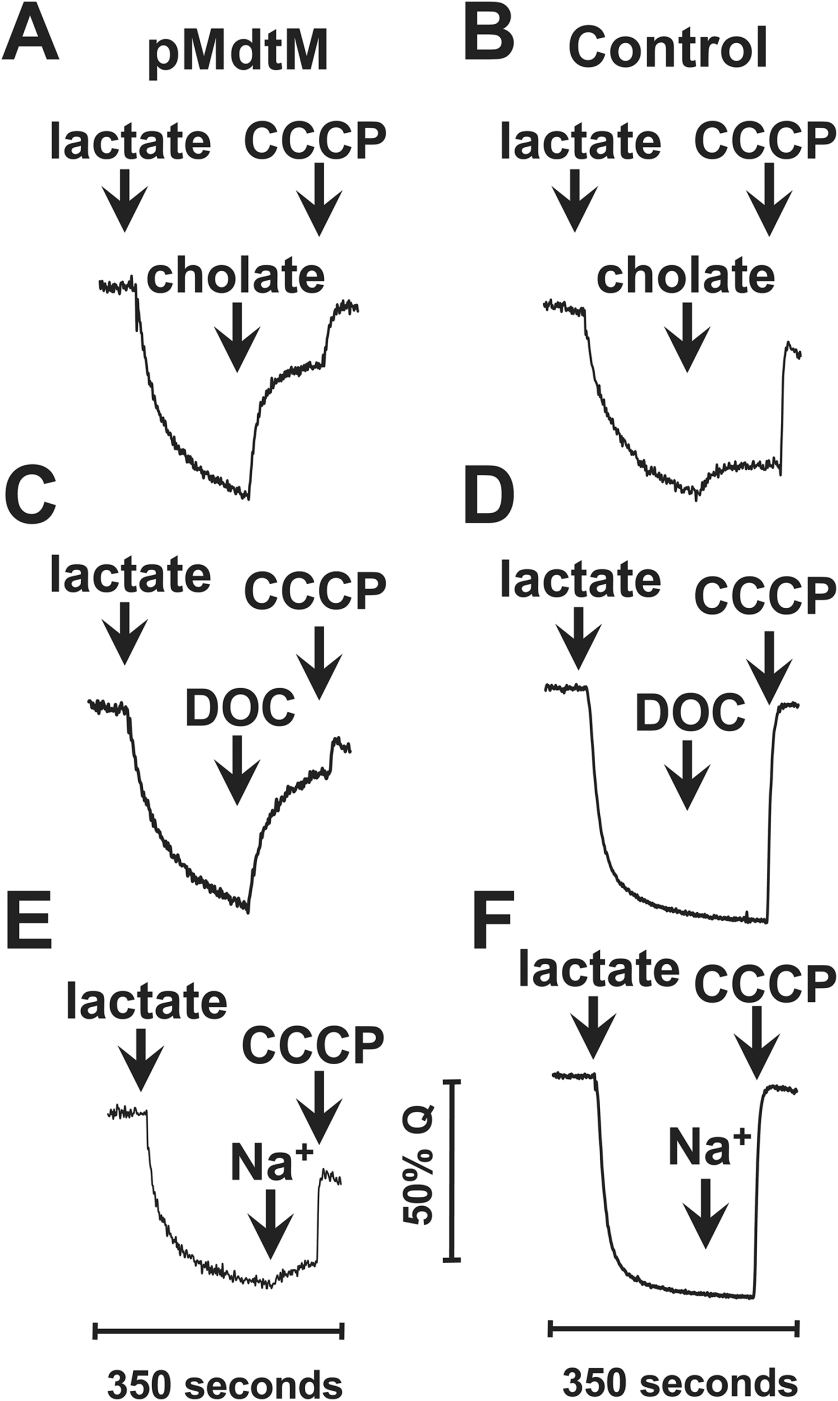
MdtM-dependent bile salt/H^+^ exchange in inverted vesicles at pH 7.2.Measurements of ΔpH were performed by monitoring the fluorescence quench/dequench of acridine orange upon addition of bile salts to inverted vesicles of *E . coli* TO114 cells that overproduced recombinant wild-type MdtM (left-hand trace of each panel) or, as a control, the dysfunctional D22A mutant (right-hand trace of each panel). Sodium cholate (A and B) or sodium deoxycholate (C and D) at final concentrations of 2.5 mM and 2 mM, respectively, was added to vesicles as indicated to initiate the transport reaction. Additional control assays were performed by the addition of sodium gluconate (Na^+^) to the inverted vesicles (E and F). Respiration-dependent generation of ΔpH (acid inside) was established by addition of lactate to 2 mM, and CCCP at a final concentration of 100 μM was used to dissipate ΔpH. The traces are representative of experiments performed in triplicate on at least two separate preparations of inverted vesicles. The fluorescence scale (50% Q) represents 50% of the initial acridine orange fluorescence signal prior to addition of lactate.

The binding affinity assays revealed that MdtM is capable of binding cholate and deoxycholate. However, evidence of binding cannot be construed as evidence of subsequent efflux. To show experimentally that MdtM can actively mediate bile salt/H^+^ antiport across the cytoplasmic membrane, transport assays on inverted membrane vesicles generated from antiporter-deficient *E. coli* TO114 cells that overproduced wild-type transporter were performed by measuring the fluorescence dequenching of the pH-sensitive fluorophore acridine orange upon addition of bile salts to the assay mixture (Figs 4 and S5). The presence of RND-type efflux proteins in the TO114 cells was not a confounding factor in the assays because those particular transporters are rendered dysfunctional during vesicle production, probably due to disruption of their quaternary structure (Thanassi *et al*., 1997). A series of initial experiments (data not shown) were performed to determine the concentration of each bile salt that gave the optimal fluorescence dequench signal; these concentrations were found to be 2.5 mM and 2.0 mM for sodium cholate and sodium deoxycholate respectively. Control experiments with vesicles containing the MdtM D22A mutant (Fig. 4B, D and F; Fig. S5B, D and F) support our argument that the fluorescence dequench measured in vesicles enriched with wild-type MdtM was specific to that transporter activity and not due to action of the bile salts on the vesicular membrane.

In assays performed at pH 7.2 (Fig. 4) addition of d-lactate to energize the vesicle membranes caused a rapid quench of the acridine orange fluorescence signal as protons were pumped by respiratory chain proteins into the vesicular lumen to generate a ΔpH (acid inside). Addition of bile salt to inverted vesicles enriched with recombinant wild-type MdtM resulted in a rapid dequenching of the acridine orange steady-state fluorescence (Fig. 4A and C). The observed dequench of the acridine orange fluorescence signal originated from alkalinization of the vesicular lumen as protons were pumped out to drive active uptake of the added bile salt, thus implying that MdtM was responsible for catalysing both cholate/H^+^ and deoxycholate/H^+^ exchange reactions. Support for the contention that the observed dequench was due exclusively to MdtM-mediated bile salt/H^+^ antiport came from assays performed on control vesicles generated from cells that overexpressed the dysfunctional MdtM D22A mutant (Fig. 4B and D). In these assays, addition of 2.0 mM sodium deoxycholate to the vesicles at the time indicated had no effect on the fluorescence signal (Fig. 4D). Addition of 2.5 mM sodium cholate to the same control vesicles, however, resulted in small but reproducible dequench of the fluorescence (Fig. 4B) that probably originates from the activity of chromosomally encoded MdtM, or from other transporters that possess cholate transport activity under the experimental conditions employed and whose role in cholate efflux is not yet described. Assays performed at pH 6.0 (Fig. S5) gave similar results to those performed at pH 7.2, except that the magnitude of the fluorescence dequench upon addition of bile salt was more pronounced in assays performed at the latter pH value.

In all the assays performed, addition of the uncoupler CCCP resulted in complete dissipation of the ΔpH across the vesicle membrane, as revealed by an instantaneous and essentially maximal dequenching of the fluorescence signal; this not only validated the assertion that the initial fluorescence dequench observed upon the addition of the bile salts was due to MdtM-catalysed bile salt/H^+^ exchange activity driven by the electrochemical gradient, but also confirmed that the inverted vesicles remained intact and maintained integrity throughout the life of the assays.

MdtM possesses an Na^+^/H^+^ antiport activity that is readily apparent at alkaline pH values, and this activity competes with the multidrug efflux role of the transporter (Holdsworth and Law, 2013). MIC measurements performed at pH 8.5 suggested that the same Na^+^/H^+^ antiport activity effectively inhibited the bile salt efflux function of MdtM (Table S1). Therefore, to exclude MdtM-mediated Na^+^/H^+^ exchange as a source of the acridine orange fluorescence dequench that occurred upon addition of the sodium salts of cholate and deoxycholate to the inverted vesicles, a further set of control assays was performed. In these assays, again performed at pH 7.2 (Fig. 4E and F) and pH 6.0 (Fig. S5E and F), 2.5 mM sodium gluconate was added at the time indicated to lactate-energized inverted vesicles produced from TO114 cells that overexpressed wild-type MdtM, or its dysfunctional D22A mutant, from plasmidic DNA. There was no measureable dequench of the respiration-induced acridine orange fluorescence quench in response to added sodium in both the test and control vesicles at pH 6.0 (Fig. S5E and F). However, at pH 7.2, addition of the monovalent cation to vesicles containing wild-type MdtM caused a small dequench (< 20% of that observed in vesicles that had bile salts added) of the fluorescence (Fig. 4E) due to residual Na^+^/H^+^ antiport activity of MdtM at the slightly alkaline pH (Holdsworth and Law, 2013). Therefore, the results of the acridine orange fluorescence quench/dequench assays clearly supported an MdtM-dependent bile salt/H^+^ antiport driven by the transmembrane electrochemical gradient.

### MdtM-catalysed bile salt/H^+^ antiport is electrogenic

**Figure 5:**
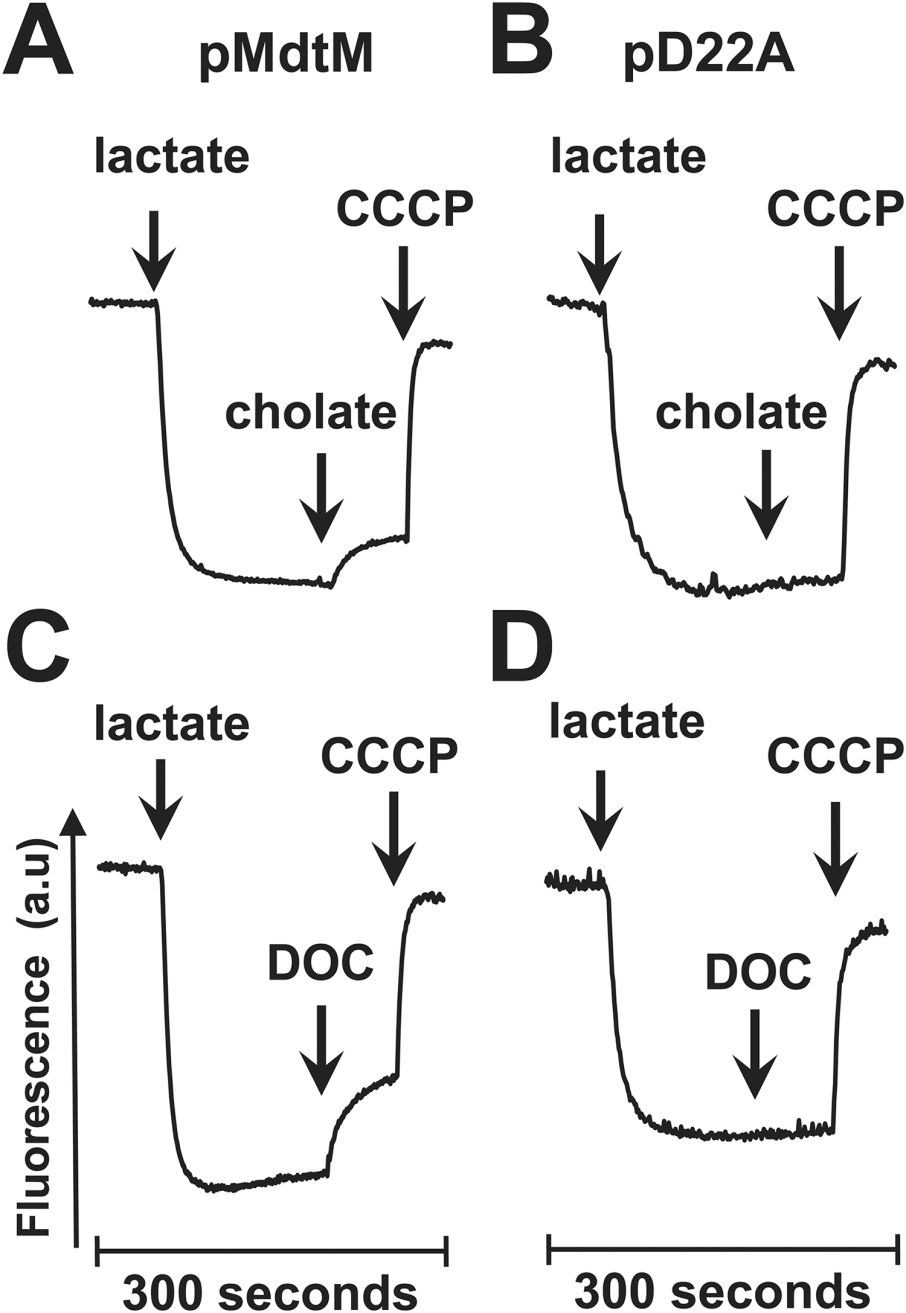
Electrogenicity of MdtM-catalysed bile salt/H^+^ antiport.Electrogenicity of MdtM-catalysed bile salt/H^+^ antiport at pH 7.2 as probed by Oxonol V fluorometry of inverted vesicles of *E . coli* TO114 cells transformed with pMdtM (left-hand traces) or, as a negative control, pD22A (right-hand traces). Respiration-dependent formation of Δψ was initiated by addition of 2 mM lactate at the time indicated and antiport was initiated by addition of sodium cholate (A and B) or sodium deoxycholate (C and D) at final concentrations of 2.5 mM and 2 mM, respectively, at the time indicated. Vesicles were depolarized by addition of CCCP to 100 μM.

To determine whether MdtM catalyses electrogenic transport by utilizing the transmembrane electrical potential (Δψ) as the driving force, inverted vesicles were produced from TO114 cells transformed with pMdtM or pD22A and assayed for electrogenicity in a chloride-free and potassium-free buffer using the Δψ-sensitive fluorophore Oxonol V. Addition of lactate to energize vesicles buffered to pH 7.2 resulted in the generation of a respiratory Δψ, as evidenced by a rapid quench of the Oxonol V fluorescence signal (Fig. 5). The addition of 2.5 mM cholate to the inverted vesicles enriched with wild-type MdtM resulted in a partial depolarization of Δψ, represented as a dequenching of the Oxonol V fluorescence, as the Δψ was consumed by the MdtM-mediated bile salt/H^+^ transport reaction (Fig. 5A). A similar response was detected upon the addition of 2.0 mM deoxycholate to wild-type MdtM-containing vesicles (Fig. 5C). In each of the assays, addition of the protonophore CCCP at the time indicated resulted in almost complete dissipation of Δψ. Addition of cholate to negative control vesicles enriched with dysfunctional MdtM D22A resulted in a perceptible dequench (Fig. 5B), probably arising from residual electrogenic Na^+^/H^+^ antiport activity of MdtM (Holdsworth and Law, 2013). In contrast, addition of the sodium salt of deoxycholate to negative control vesicles resulted in no detectable depolarization of the transmembrane potential (Fig. 5D). In this instance, the lack of any dequench signal was probably due to the fact that MdtM possesses low affinity for Na^+^ cations (Holdsworth and Law, 2013), and the 2.0 mM concentration of Na^+^ cations in the assay was insufficient to elicit any measureable electrogenic transport. The results of assays performed at pH 6.0 (Fig. S6) were similar to those observed for assays performed at pH 7.2, except that addition of cholate to negative control vesicles enriched with dysfunctional MdtM D22A mutant resulted in no detectable depolarization (Fig. S6B).

Although the set of experiments described above showed that MdtM-dependent antiport was Δψ-consuming, from which it was inferred to be electrogenic, to provide assurance that it was bile salt/H^+^ antiport and not Na^+^/H^+^ antiport activity that was being measured, additional control experiments were performed to test the effects of adding sodium gluconate in place of the bile salts, and by adding the ionophores nigericin (to selectively dissipate ΔpH) and valinomycin (to selectively dissipate Δψ) to inverted vesicles that overproduced wild-type MdtM (Fig. S7). As shown in Fig. S7C, no response of the Oxonol V fluorescence signal was observed upon addition of 2.5 mM sodium gluconate to the vesicles at pH 6.0. However, as expected, due to the electrogenic Na^+^/H^+^ antiport activity of MdtM at alkaline pH (Holdsworth and Law, 2013), a small dissipation of Δψ was recorded in the assay performed at pH 7.2 (Fig. S7A). Addition of valinomycin to abolish the Δψ (Fig. S7A), or the protonophore CCCP to abolish both components of the electrochemical gradient (Fig. S7C) to the vesicles at the times indicated caused a rapid and complete dequench of the Oxonol V fluorescence. In a final set of controls, nigericin was added to inverted, wild-type MdtM-containing vesicles pre-incubated with 50 mM potassium gluconate (Fig. S7B and D). In the assay performed at pH 7.2 addition of the ionophore resulted in a small increase in the magnitude of Δψ (observed on the assay trace in Fig. S7B as a further quench in the Oxonol V fluorescence signal) as ΔpH was converted to Δψ by the electroneutral K^+^/H^+^ exchange activity of the ionophore (Padan *et al*., 1976). However, in the same assay performed at pH 6.0 (Fig. S7D) no such conversion was observed, probably due to activity of other transporters that could maintain the ΔpH in the more acidic environment. As observed in the previous assays, valinomycin or CCCP caused a complete dissipation of Δψ. All the control experiments provided evidence that the vesicles had retained integrity and were therefore able to maintain an electrochemical potential across the membrane during the assay lifetime, and also established that the nature of MdtM-catalysed cholate/H^+^ and deoxycholate/H^+^ antiport is electrogenic.

## Discussion

Consistent with a prior hypothesis that a significant part of bile salt efflux is performed by a membrane transporter system(s) other than AcrAB–TolC and EmrAB–TolC (Thanassi *et al*., 1997), the results presented here provide evidence that the *E. coli* single-component MFS transporter MdtM, originally characterized as a multidrug efflux protein (Holdsworth and Law, 2012a, b), functions in this role.

MIC measurements demonstrated that although not essential for resistance of *E. coli* to cholate and deoxycholate, MdtM does make a significant contribution to it (Table 1). Transcript levels of mRNA encoding MdtM, however, did not increase in response to cholate or deoxycholate exposure; this was not unexpected since other single-component transporters that function physiologically in bile salt efflux in other enteric bacterial species are not upregulated at the level of mRNA in response to bile challenge (Bron *et al*., 2006; Whitehead *et al*., 2008; Hernandez *et al*., 2012).

The notion that MdtM plays a substantial role in bile salt resistance in *E. coli* was reinforced by IC_50_ measurements made at two physiologically relevant pH values (Fig. 1). These measurements clearly showed that complementation of the Δ*mdtM* mutant with plasmidic DNA (pMdtM) encoding wild-type MdtM under control of an arabinose-inducible promoter recovered a bile salt-resistant phenotype. The calculated IC_50_ values also revealed that, at pH 7.2, wild-type *E. coli* was significantly less tolerant of deoxycholate than of cholate, an observation consistent with the hydrophobicity of the bile salts tested, which is higher for deoxycholate than for cholate, and hence, reflects the capacity of each compound to permeate the phospholipid membrane and enter the cell cytoplasm (Heuman *et al*., 1989; Kamp *et al*., 1993). In contrast, at the more acidic pH of 6.0, there was no significant difference in susceptibility to the two different bile salts; however, at this pH the IC_50_ for cholate was only about 60% of that measured at pH 7.2. These observations can be rationalized if consideration is given to the ionization state of the bile salts at each pH. Cholate and deoxycholate are weak acids with pKa values of 6.4 and 6.6 respectively (Budavari, 1996; Yokota *et al*., 2000). Therefore, at pH 7.2, both would exist in predominantly anionic form, with only ∼ 13% of the deoxycholate and ∼ 20% of the cholate in an undissociated, lipophilic form that can diffuse more easily across the membrane bilayer. At pH 6.0, the prevalence of the protonated, electroneutral state of each bile salt means that more molecules are in a form that can readily enter the cell cytoplasm where they can build up to potentially cytotoxic concentrations. Although this may explain the approximately twofold difference between the IC_50_ values for cholate measured at pH 7.2 and pH 6.0, it does not explain the lack of a similar apparent decrease in resistance towards deoxycholate. This may be a consequence of the more efficient partitioning of deoxycholate into the cell cytoplasm, which means that at pH 7.2 the machinery that comprises the bile salt ‘effluxome’ is already saturated. An alternative explanation could lie in the pH dependence of substrate binding to the transporter protein(s) involved in cholate and deoxycholate efflux due to different ionization state(s) of the substrate binding site(s) at each pH, or the specificity of the same protein(s) for different charged states of the bile salt cargo.

Substrate binding studies that exploited intrinsic tryptophan fluorescence of purified MdtM protein in detergent solution established that both bile salts tested bound to purified MdtM with micromolar affinity (Fig. 3), an observation in agreement with the transporter functioning as a high-affinity bile salt efflux protein. The lower apparent affinity (K_d_^app^) of MdtM for deoxycholate compared to cholate may help explain the results of the IC_50_ assays described above; the less efficient binding by MdtM of deoxycholate that has diffused into the cytoplasm would make the cells more susceptible to the cytotoxic effects of this particular bile salt. Furthermore, the affinity of each bile salt for MdtM displayed a striking pH-dependence that resulted in reduced binding at lower pH. Analysis of the circular dichroism spectra of MdtM at pH 6.0 and pH 7.2 confirmed that the reduced affinity for each substrate at the acidic pH did not arise due loss of structural integrity of the DDM-solubilized transporter. Instead, the reduced affinity at acidic pH suggests that a change in the protonation state of individual amino acid side-chains that are important for MdtM function affects substrate binding; just such a competition for binding between protons and antimicrobial substrate has been demonstrated in another MFS drug/proton antiporter, MdfA, a close *E. coli* homologue of MdtM (Fluman *et al*., 2012).

Single-component, secondary active multidrug antiporters like MdtM cannot efflux antimicrobials completely out of the bacterial cell; they can only catalyse their transport from the cytoplasm into the periplasmic space. In the absence of an additional efflux system capable of catalysing transport from the periplasm into the extracellular environment, the cytotoxins would simply build up in the periplasmic space and leak back into the cytoplasm. Evidence suggests that the single-component multidrug/proton antiporters do not act unilaterally (Lee *et al*., 2000), and that a functional synergism between them and tripartite transporters such as AcrAB–TolC exists to confer levels of resistance to antimicrobials that would otherwise not be achievable. Indeed, it has been shown before that both MdtM and MdfA interact synergistically with a tripartite efflux system(s), most probably AcrAB–TolC, to provide *E. coli* with an enhanced efflux activity to rid the cell of antimicrobials (Tal and Schuldiner, 2009; Holdsworth and Law, 2012a, b). Comparison of IC_50_ measurements for cholate and deoxycholate made on the *acrB* chromosomal deletion mutant that expressed dysfunctional MdtM, and on the Δ*acrB* mutant complemented with pMdtM, suggested just such synergism exists between MdtM and AcrAB–TolC for efflux of bile salts. MdtM and AcrAB–TolC appear to operate in series – and therefore their effect on resistance is multiplicative – to provide an effective mechanism of flushing bile salts completely out of the cell. Disruption of one part of this dual-stage efflux system increases the susceptibility of *E. coli* to antimicrobials, with hypersensitivity especially evident upon dysfunction of the tripartite efflux system. This dual-stage efflux likely represents a general mechanism for multidrug resistance in bacteria.

Although most characterized, substrate-specific secondary active transporters catalyse only one or other of electrogenic or electroneutral transport, the ability of their multidrug efflux counterparts to transport substrates of different charges means that secondary active multidrug transporters possess capacity to catalyse both types of electrically distinct transport reaction (Lewinson *et al*., 2003). In the case of bile salt efflux, the electrogenicity of the MdtM-mediated transport reaction raises interesting questions as to the electronic nature of the transported substrate and the stoichiometry of antiport. At the relatively alkaline pH of between 7.4 and 7.8 that exists in the *E. coli* cell cytoplasm (Padan *et al*., 1976) about 90% of the cholate and deoxycholate molecules present in this environment would exist in anionic form. Therefore, in order to be transported across the inner membrane in a neutral form, they must be associated either with a proton or with a monovalent cation such as Na^+^. Although the assays described here do not permit an analysis of the stoichiometry of MdtM-catalysed bile salt/H^+^ antiport, we favour a scenario whereby one proton is exchanged for one cholate or deoxycholate molecule.

The bile salt efflux role of MdtM highlights the extraordinary capacity of single-component multidrug transporters of the MFS to function in a diversity of physiologically relevant roles. It is probable that the physiological roles of MFS multidrug transporters contribute to their conservation in bacterial genomes and, consequently, the spread of multidrug resistance. Notably, the capacity of these proteins to function effectively in the disparate and harsh environments encountered in the human gastrointestinal tract provides a clue as to why *E. coli* and other Gram-negative bacterial infections are challenging to treat with currently available antibiotics.

## Experimental procedures

All growth media, antibiotics and chemicals were purchased from Sigma-Aldrich (Poole, Dorset, UK) unless stated otherwise.

### Bacterial strains and plasmids

*Escherichia coli* BW25113 {*rrnB3* Δ*lacZ4787 hsdR514* Δ(*araBAD*)567 Δ*(rhaBAD)*568 *rph-1*} (Datsenko and Wanner, 2000) and its Δ*mdtM,* Δ*acrB*, Δ*emrB* and Δ*ompF* single-deletion mutants were obtained from the Keio collection (National BioResource Project, Japan) (Baba *et al*., 2006) and used for MIC assays. The Δ*mdtM* and Δ*acrB* deletion mutants were used as the background strains for determining IC_50_ values of cells expressing wild-type *mdtM* (pMdtM) or, as a control, dysfunctional MdtM D22A (pD22A) mutant from pBAD/*Myc*-His A vector (Life Technologies, Paisley, UK) (Holdsworth and Law, 2012a). Ethidium bromide efflux assays used the outer membrane permeability mutant UTL2 strain of *E. coli* (Beja and Bibi, 1996). For production of inverted vesicles used in transport assays, *E. coli* TO114 complemented with pMdtM or pD22A was used. The TO114 strain is deficient in the Na^+^/H^+^ antiporters NhaA and NhaB, and the K^+^/H^+^ antiporter ChaA. This strain was chosen specifically to abrogate any potential interference from NhaA and NhaB Na^+^/H^+^ antiport activity (Holdsworth and Law, 2013). Overproduction of wild-type and mutant MdtM for purification and subsequent use in substrate-binding assays was performed in *E. coli* LMG194 (KS272 Δ*ara714 leu*::Tn*10*) transformed with the appropriate plasmid (Holdsworth and Law, 2012a).

### Construction of MdtM mutants

MdtM W309F and W309A mutants were produced using the QuikChange Lightning site-directed mutagenesis kit (Agilent Technologies) with pMdtM as the template, and the following 5′–3′ PCR primers (W309F forward: tcgccgcacgtcttcctgtggtcggtgc; W309F reverse: gcaccgaccacaggaagacgtgcggcga; W309A forward: gtcgccgcacgtcgcgctgtggtcggtg; W309A reverse: caccgaccacagcgcgacgtgcggcgac). The fidelity of each mutant construct was verified by DNA sequence analysis.

### Determination of MIC of bile salts

MICs of the sodium salts of cholate and deoxycholate were determined using serial twofold dilutions of each bile salt from a 128 mg ml^−1^ stock on solid Luria–Bertani (LB) agar plates, each inoculated with 10^4^ colony-forming units (cfu) of the strain to be tested. The plates were incubated overnight at 37°C prior to ocular inspection for colony growth.

### Quantitative PCR analysis of *mdtM* transcript levels

To test for the effects of bile salt on transcript levels of MdtM, total RNA was extracted from cell pellets prepared from duplicate 1 ml samples of mid-log phase, wild-type *E. coli* BW25113 with or without 20 mM sodium cholate, and taken at t = 0 min, t = 15 min and t = 30 min. RNA (1 μg) was treated with DNase (Turbo DNA Free, Life Technologies) before reverse-transcription to cDNA. Transcriptional changes in *mdtM* between cholate-treated and untreated samples were assessed using real-time quantitative PCR analysis of these cDNAs, with changes expressed relative to a reference transcript (*rrsB*, 16S rRNA). PCR primers were designed against GenBank-derived *rrsB* and *mdtM* sequences (*rrsB* forward: agagcaagcggacctcataa; rrsB reverse: aacgtattcaccgtggcatt; *mdtM* forward: gcctgggatcattaatgtgg; *mdtM* reverse: agccactgtaacgccatacc). Following collection of Ct and efficiency values using RotorGene software, transcriptional changes in *mdtM* were determined using the Augmented ΔΔCt method (Pfaffl, 2001).

### Determination of IC_50_ of cholate and deoxycholate

To test the contribution of MdtM to intrinsic resistance against bile salts, growth of *E. coli* cells was measured in LB liquid medium that contained varying concentrations of the sodium salts of cholate or deoxycholate at pH values of 7.2 and 6.0. The pH of the medium was buffered by 70 mM 1,3-bis[tris(hydroxymethyl)-methylamino] propane (BTP) and pH was adjusted by HCl. Assays were performed based on a previously described method (Holdsworth and Law, 2012b).

### Whole-cell ethidium bromide transport assays

The effect of addition of 3.0 mM sodium cholate and 3.0 mM sodium deoxycholate on ethidium bromide (EtBr) efflux by *E. coli* outer membrane permeability mutant UTL2 cells enriched with MdtM was determined using a method described before (Holdsworth and Law, 2013) except that assays were performed at pH 6.0 to abrogate interference by MdtM-catalysed Na^+^(K^+^)/H^+^ antiport that occurs at alkaline pH. To test that bile salt had not compromised cell membrane integrity, UTL2 cells used to investigate the inhibitory effects of 3.0 mM sodium cholate on the EtBr efflux activity of MdtM were removed from the cuvette, washed twice in assay buffer to remove CCCP, then re-energized by addition of glucose.

### Measurement of proton-driven antiport

Assays of bile salt/H^+^ antiport were conducted by measuring the fluorescence quenching/dequenching of the pH-sensitive indicator acridine orange upon addition of either 2.5 mM cholate or 2.0 mM deoxycholate to energized inverted membrane vesicles generated from antiporter-deficient *E. coli* TO114 cells that overproduced recombinant wild-type MdtM. Inverted vesicles of TO114 cells that overproduced dysfunctional MdtM from pD22A were used as controls. Transport measurements were performed as described before (Holdsworth and Law, 2012b) at pH values of 7.2 and 6.0. Control experiments to ensure any observed fluorescence dequench was not a result of MdtM-mediated Na^+^/H^+^ antiport were performed by addition of 2.5 mM sodium gluconate instead of bile salt. All experiments were performed in triplicate on at least two separate preparations of inverted vesicles.

### Measurement of the electrogenicity of bile salt/*H*^+^ antiport

The Δψ-sensitive fluorophore Oxonol V [bis-(3-phenyl-5-oxoisoxazol-4-yl)pentamethine oxonol] (Cambridge Bioscience, Cambridge, UK) was used to determine if the MdtM-mediated antiport observed in the previous experiments was electrogenic. Inverted vesicles were produced from TO114 cells transformed with pMdtM or pD22A as described previously (Holdsworth and Law, 2013), except that the vesicle resuspension buffer was made Cl^−^-free by substitution of the 140 mM choline chloride component with 280 mM sorbitol and by using H_2_SO_4_ rather than HCl to adjust buffer pH. Vesicles (0.5 mg ml^−1^ membrane protein) were added to assay buffer (10 mM BTP, 5 mM MgSO_4_, 5 μM Oxonol V) that had its pH adjusted to 7.2 or 6.0. Electrogenic antiport activity was estimated on the basis of its ability to dissipate the established Δψ (recorded as a dequenching of the fluorescence signal) in response to addition of 2.5 mM sodium cholate or 2.0 mM sodium deoxycholate to vesicles at the times indicated. As a control experiment, to ensure that there was no interference from MdtM-mediated Na^+^/H^+^ antiport, 2.5 mM sodium gluconate was substituted for bile salt. As a further control, 1 μM of the ionophore nigericin (which at low concentrations selectively consumes ΔpH in the presence of K^+^ via electroneutral K^+^/H^+^ exchange) was added to vesicles of TO114 cells transformed with pMdtM. These vesicles were incubated in assay buffer that contained 50 mM K^+^ gluconate, and valinomycin (5 μM) was added to selectively abolish Δψ.

### Purification of MdtM

Wild-type and mutant protein for use in substrate binding assays and circular dichroism (CD) spectroscopy studies was homologously overexpressed in *E. coli* LMG194 cells and purified following the protocol described previously (Holdsworth and Law, 2012a). Purified protein was placed on ice and used immediately for substrate binding studies. Concentrated MdtM solution for use in CD studies was diluted down with an imidazole- and NaCl-free buffer to minimize interference from those compounds with the spectroscopic measurements.

### Substrate binding assays

Substrate-binding affinity of purified, wild-type MdtM for the sodium salts of cholate and deoxycholate was determined at pH 7.2 and pH 6.0 using intrinsic tryptophan fluorescence quenching of protein in detergent solution based on the protocol described previously (Holdsworth and Law, 2012b). Control assays that investigated binding of cholate to the W309F and W309A MdtM mutants were performed at pH 7.2.

### Circular dichroism (CD) spectroscopy

Far UV circular dichroism spectra were recorded using a JASCO J-810 spectropolarimeter (Jasco UK). MdtM samples were analysed at pH 6 and pH 7.2 using a 5.3 μM protein solution in a 0.02 cm pathlength quartz cuvette. A suitable buffer baseline was collected and subtracted from each protein spectrum. Corrected data were expressed in terms of mean residue ellipticity (degrees cm^−2^ dmol^−1^). The online CD secondary structure analysis server Dichroweb (Whitmore and Wallace, 2004) and the CONTIN procedure Reference set 4 (Provencher and Glockner, 1981) was used to analyse the CD data.

### Western blot analysis of recombinant MdtM

Estimation of expression levels of recombinant wild-type and D22A mutant MdtM in transformed BW25113 Δ*mdtM* and BW25113 Δ*acrB* cells grown at pH 7.2 and pH 6.0 was performed by Western blot analysis following a protocol described before (Holdsworth and Law, 2013).

## Competing interests

The authors declare no competing interests.
