# Supplementary material for: A single-component multidrug transporter of the major facilitator superfamily is part of a network that protects E scherichia coli from bile salt stress
Source: Mol Microbiol. 2014 Apr 13;92(4):872–84. doi: 10.1111/mmi.12597 (PMC4235344; doi:10.1111/mmi.12597)
Supplement: Supplementary file 1 [file mmi0092-0872-SD1.pdf]

Supplementary Material

Table S1. MIC of bile salts in isogenic strains of *E. coli* BW25113 at pH 8.5. At alkaline pH, wild type cells are 4-fold more susceptible than cells grown at neutral pH (Table 1) to the toxic effects of bile salts, and deletion of chromosomal *mdtM* has no apparent effect on bile salt resistance.

Strain	MIC (mg/ml) of:	
	Na^+ cholate	Na^+ deoxycholate
Wild type	32	32
Δ*mdtM*	32	32

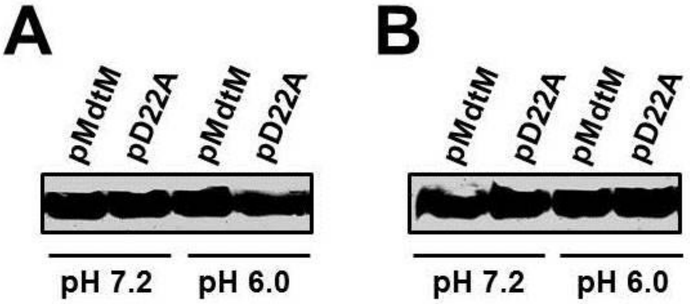

Figure S1. Western blot analysis of expression levels of recombinant wild type (pMdtM) and D22A mutant (pD22A) MdtM overproduced by **(A)** *E. coli* BW25113 Δ*mdtM* single-deletion mutant cells grown at pH 7.2 and pH 6.0, and **(B)** by Δ*acrB* chromosomal deletion mutant BW25113 cells grown at two different pH values. Each lane contained 10 μg of membrane protein.

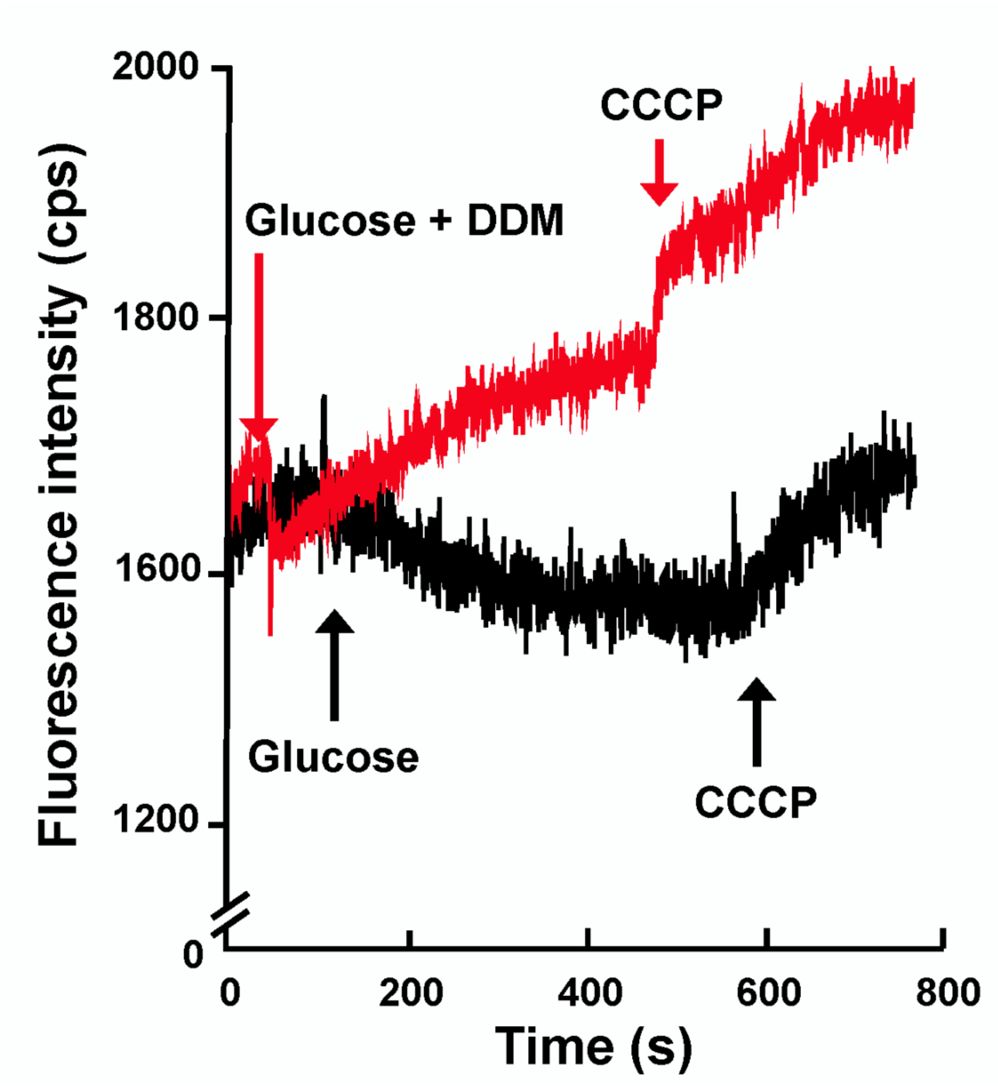

Figure S2. Whole cell EtBr efflux assay control experiments. Addition of 0.1 mM DDM detergent along with the glucose used to energise *E. coli* UTL2 cells expressing recombinant wild-type MdtM and loaded with EtBr resulted in an increase in the fluorescence emission (red trace). In contrast, the fluorescence of re-energised UTL2 cells that had previously been exposed to 3 mM sodium cholate (black trace) was steadily quenched until addition of CCCP at the time indicated. All assays were performed at pH 6.0. Fluorescence intensity was measured in counts per second (cps).

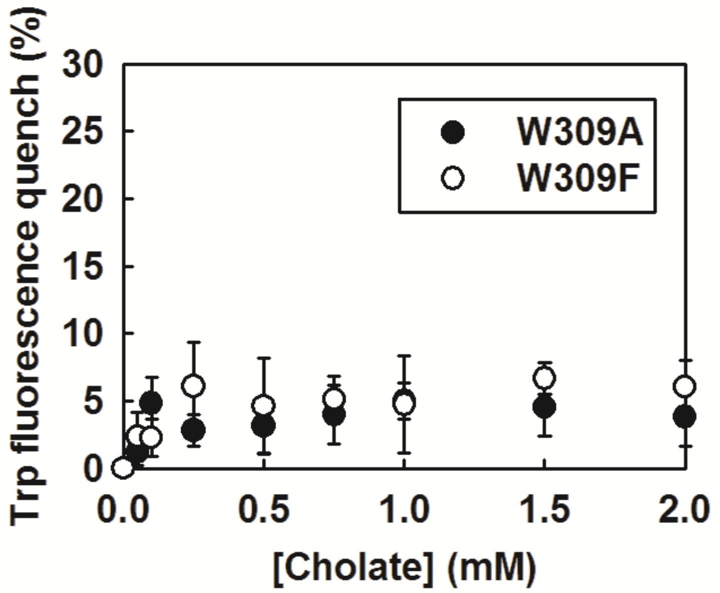

Figure S3. Percentage intrinsic fluorescence quenching of purified W309F and W309A MdtM in DDM solution as a function of cholate concentration at pH 7.2. Data points and error bars represent the mean±SD of three individual measurements.

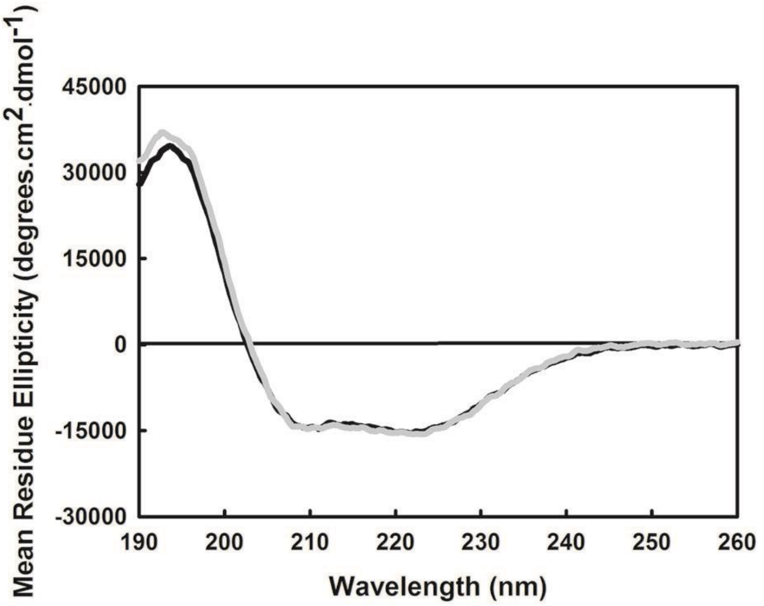

Figure S4. Circular dichroism spectra of purified, DDM detergent-solubilised MdtM at pH 7.2 (grey trace) and pH 6.0 (black trace).

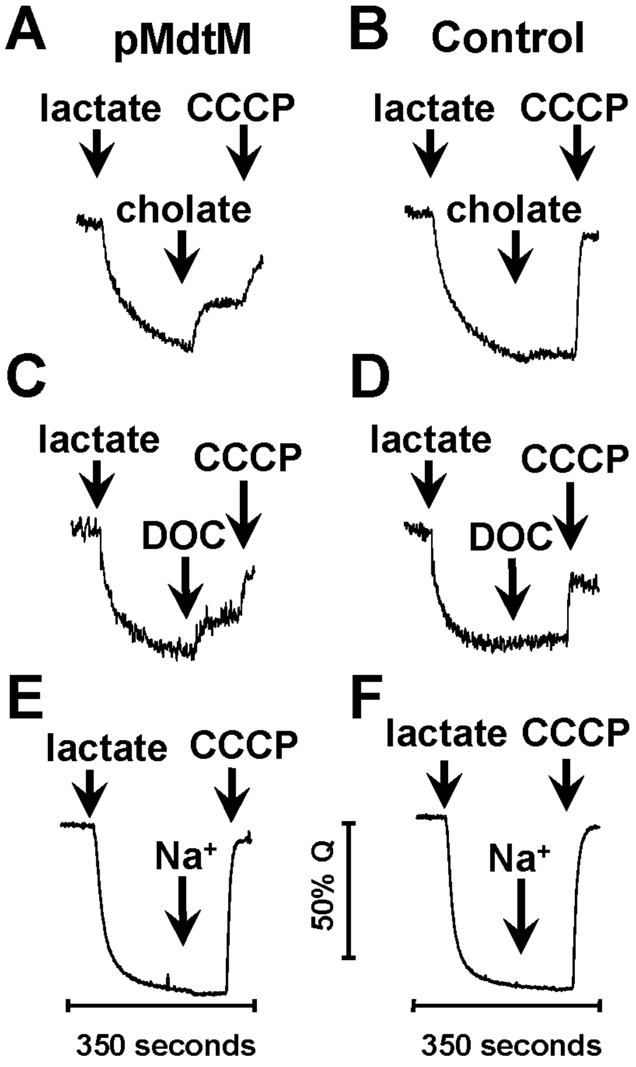

Figure S5. MdtM-dependent bile salt/H^+ exchange in inverted vesicles at pH 6.0. Measurements of ΔpH were performed by monitoring the fluorescence quench/dequench of acridine orange upon addition of bile salts to inverted vesicles that harboured recombinant wild type MdtM **(A, C & E)** or, as a control, the dysfunctional D22A mutant **(B, D & F)**. Sodium cholate **(A & B)** or sodium deoxycholate, DOC, **(C & D)** was added to vesicles as indicated to initiate the transport reaction. Additional control assays were performed by the addition of sodium gluconate (Na^+) to the inverted vesicles (**E & F**). Respiration-dependent generation of ΔpH (acid inside) was established by addition of lactate. Addition of CCCP at the time indicated completely dissipated ΔpH. The traces are representative of experiments performed in triplicate on at least two separate preparations of inverted vesicles. The fluorescence scale (50% Q) represents 50% of the initial acridine orange fluorescence signal prior to addition of lactate.

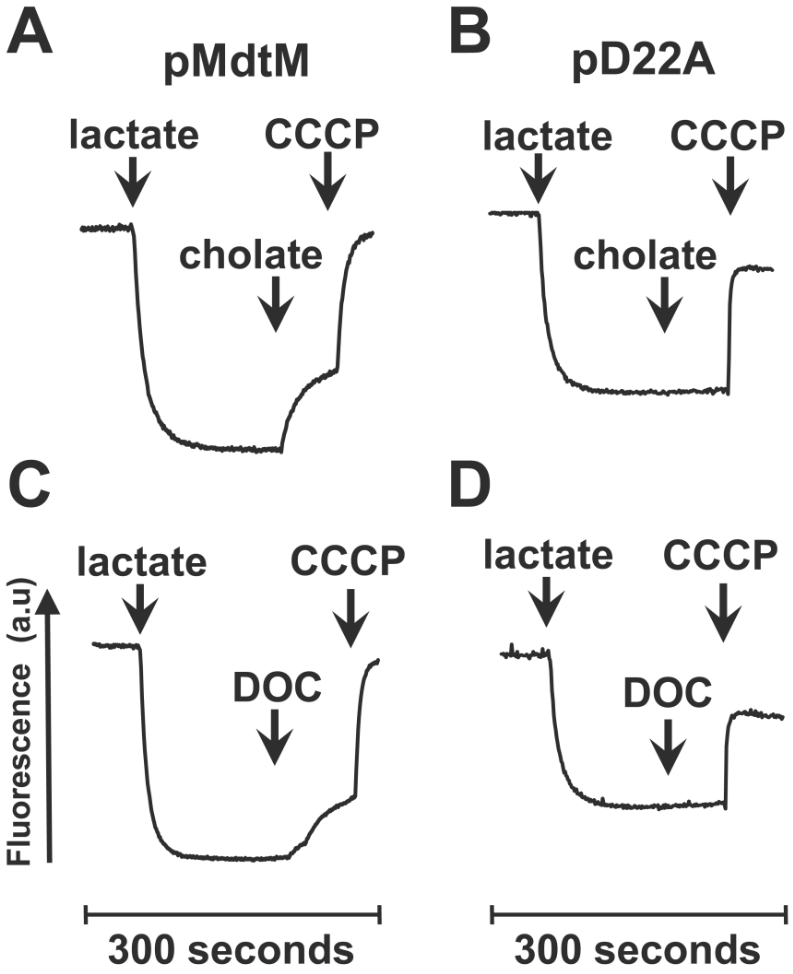

Figure S6. Electrogenicity of MdtM-catalysed bile salt/H^+ antiport at pH 6.0. Electrogenic antiport was probed by Oxonol V fluorometry of inverted vesicles generated from *E. coli* TO114 cells transformed with pMdtM (left-hand traces) or, as a negative control, pD22A (left-hand traces). Respiration-dependent formation of $\Delta\psi$ was initiated by addition of lactate at the time indicated. Once steady-state $\Delta\psi$ was achieved, antiport was initiated by addition of sodium cholate **(A & B)** or sodium deoxycholate **(C & D)** as indicated. Vesicles were depolarised by addition of CCCP as indicated.

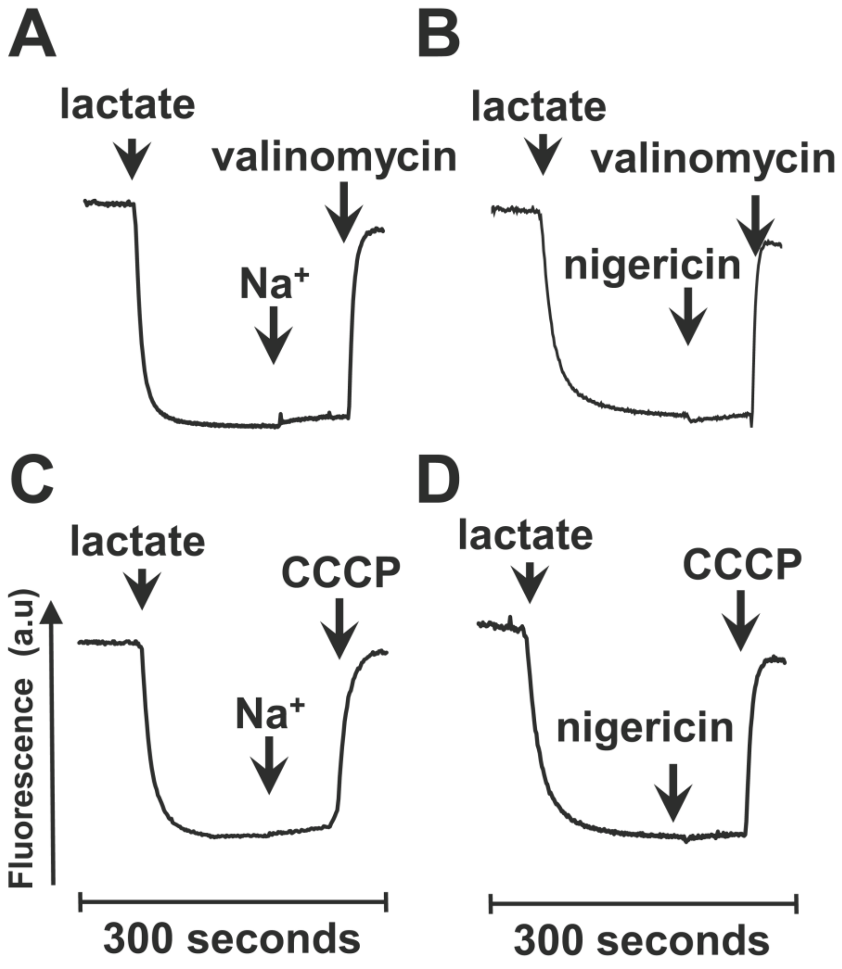

Figure S7. MdtM catalyses electrogenic bile salt/H^+ antiport. Control experiments, in which the effects of addition of sodium gluconate **(A & C)** and the ionophore nigericin **(B & D)** were probed by Oxonol V fluorometry of inverted vesicles of TO114 cells transformed with pMdtM, were performed at pH 7.2 **(A & B)** and pH 6.0 **(C & D)**. The traces shown are representative of experiments performed in triplicate on two separate preparations of inverted vesicles.
